# The tuberculosis-associated immune reconstitution inflammatory syndrome: recent advances in clinical and pathogenesis research

**DOI:** 10.1097/COH.0000000000000502

**Published:** 2018-09-15

**Authors:** Naomi F. Walker, Cari Stek, Sean Wasserman, Robert J. Wilkinson, Graeme Meintjes

**Affiliations:** a Department of Clinical Research, London School of Hygiene and Tropical Medicine, London, UK; b Wellcome Centre for Infectious Diseases Research in Africa, Institute of Infectious Disease and Molecular Medicine, University of Cape Town, Cape Town, South Africa; c Department of Clinical Sciences, Institute of Tropical Medicine Antwerp, Antwerp, Belgium; d Department of Medicine, University of Cape Town, Cape Town, South Africa; e Department of Medicine, Imperial College London; f Francis Crick Institute, London, UK

**Keywords:** HIV-1 infection, immune reconstitution inflammatory syndrome, paradoxical, tuberculosis, unmasking

## Abstract

**Purpose of review:**

Antiretroviral therapy (ART) is an essential, life-saving intervention for HIV infection. However, ART initiation is frequently complicated by the tuberculosis-associated immune reconstitution inflammatory syndrome (TB-IRIS) in TB endemic settings. Here, we summarize the current understanding highlighting the recent evidence.

**Recent findings:**

The incidence of paradoxical TB-IRIS is estimated at 18% (95% CI 16–21%), higher than previously reported and may be over 50% in high-risk groups. Early ART initiation in TB patients increases TB-IRIS risk by greater than two-fold, but is critical in TB patients with CD4 counts less than 50 cells/μl because it improves survival. There remains no validated diagnostic test for TB-IRIS, and biomarkers recently proposed are not routinely used. Prednisone initiated alongside ART in selected patients with CD4 less than 100 cells/μl reduced the risk of paradoxical TB-IRIS by 30% in a recent randomized-controlled trial (RCT) and was not associated with significant adverse effects. Effective also for treating paradoxical TB-IRIS, corticosteroids remain the only therapeutic intervention for TB-IRIS supported by RCT trial data. TB-IRIS pathogenesis studies implicate high antigen burden, innate immune cell cytotoxicity, inflammasome activation and dysregulated matrix metalloproteinases in the development of the condition.

**Summary:**

Specific biomarkers would aid in identifying high-risk patients for interventions and a diagnostic test is needed. Clinicians should consider prednisone for TB-IRIS prevention in selected patients. Future research should focus on improving diagnosis and investigating novel therapeutic interventions, especially for patients in whom corticosteroid therapy is contraindicated.

## INTRODUCTION

HIV-associated TB is common, with an estimated 1.4 million cases and 374 000 deaths annually [1]. In parts of sub-Saharan Africa, around 60% of TB patients are HIV coinfected [1]. Antiretroviral therapy (ART) is an essential, life-saving intervention for HIV, but HIV-infected patients starting ART are at high risk of tuberculosis-associated immune reconstitution inflammatory syndrome (TB-IRIS) in TB endemic settings. TB-IRIS is an acute inflammatory condition that presents with worsening, or development of new, tuberculosis disease in a patient already on TB treatment after starting ART (paradoxical TB-IRIS), or a new diagnosis of TB with a particularly acute, inflammatory presentation after starting ART (unmasking TB-IRIS). Rapid restoration of immunity after ART, with exaggerated inflammatory responses to *Mycobacterium tuberculosis* (Mtb) antigens, underlies this condition although the pathophysiology is incompletely understood.

In a systematic review, Namale *et al.*[2] collated studies published before 3 May 2014 reporting incidence, clinical features, management and outcomes of paradoxical TB-IRIS, including 40 studies, 7789 patients at risk and 1048 TB-IRIS cases. Studies were from Africa, Asia, Europe, North and South America. Here, we discuss key findings in subsequently published literature, on TB-IRIS epidemiology, outcomes, management, prevention, diagnosis and pathogenesis. We focus on paradoxical TB-IRIS, the most common form of HIV-associated IRIS and most frequently studied. Unmasking TB-IRIS is discussed in a separate section. Knowledge to date, with reference to key review articles and recent original research articles, is summarized in Table 1 [2,3^▪^,4–6,7^▪^,8,9,10^▪^,11,12^▪▪^]. 

**Box 1:**
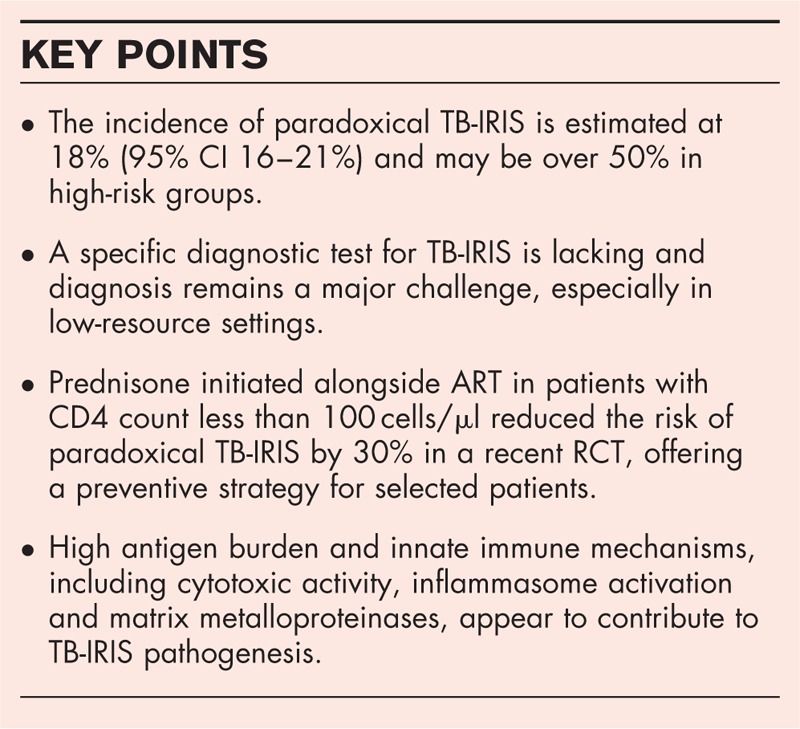
no caption available

## DEFINITIONS

**FIGURE 1:**
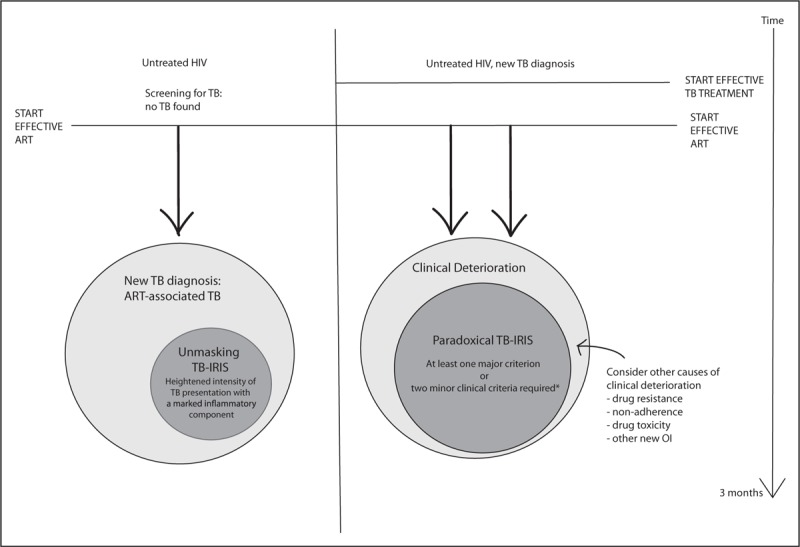
Definitions of TB-IRIS. Adapted from INSHI definition. TB-IRIS can also occur when ART is reinitiated after stopping ART and when changing from a failing regiment to a new effective ART regimen. ART, antiretroviral therapy. ^∗^Major criteria for paradoxical TB-IRIS: (i) new/enlarging LN, cold abscess or other focal tissue involvement; (ii) new/worsening radiological features of TB; (iii) new or worsening central nervous system tuberculosis; (iv) new or worsening serositis. Minor criteria for paradoxical TB-IRIS: (i) new/worsening constitutional symptoms; (ii) new/worsening respiratory symptoms; (iii) new/worsening abdominal pain and peritonitis/hepatomegaly/splenomegaly/abdominal adenopathy.

**FIGURE 2:**
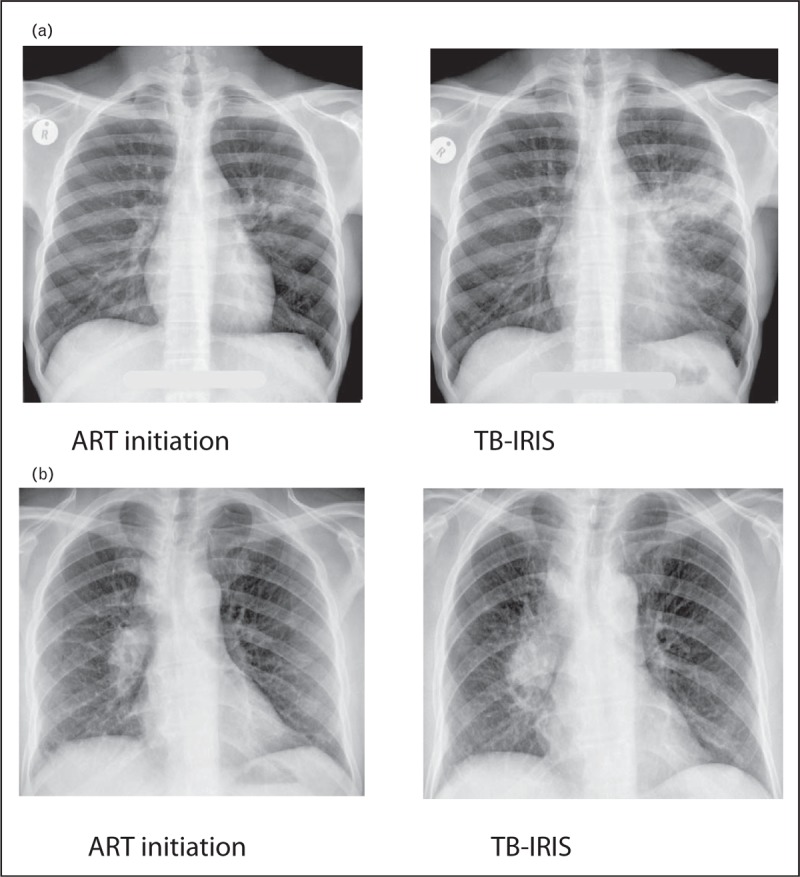
Paradoxical TB-IRIS case illustrations. Patient (a) was a 29-year-old man, with a CD4 count of 14 cells/μl, who had been on TB treatment for 1 month. He complained of loss of appetite, 4 kg weight loss and recurrence of cough and chest pain 2 weeks after starting ART. Chest radiograph shows extension of the left hilar infiltrate. His C-reactive protein had increased from 11 mg/l before the start of ART to 292 mg/l. His symptoms spontaneously resolved 2 weeks later. Patient (b) was a 36-year-old man, with a CD4 count of 73 cells/μl, who had been on TB treatment for 3 weeks. One week after starting ART, he complained about poor appetite, gradually worsening with dyspnoea, cough, night sweats, diarrhoea, vomiting and fatigue. He was tachypnoeic, with a temperature of 38^°^C. Chest radiograph showed an increase in hilar and paratracheal lymphadenopathy. His C-reactive protein had increased from 4 mg/l before the start of ART to 120 mg/l. He was started on prednisone treatment, resulting in complete resolution of his symptoms over the next few weeks.

As there are no validated diagnostic tests for TB-IRIS, diagnosis is clinical. The International Network for the Study of HIV-associated IRIS (INSHI) consensus definitions of paradoxical and unmasking TB-IRIS are the most commonly used and have been validated [13,14]. The Aids Clinical Trials Group definition (see https://actgnetwork.org/IRIS_Case_Definitions) and IMPAACT trial definition (see https://www.impaactnetwork.org for Adolescent and Paediatric cohorts) have been used in research settings. The schematic in Fig. 1 summarizes the different terms used and the relationship to TB treatment and ART initiation. The case definitions provide subclassification into confirmed and probable paradoxical TB-IRIS according to the extent in which other possible causes of symptoms have been adequately excluded. ART-associated TB is a broad term that encompasses new TB diagnoses in patients who have recently commenced ART, including unmasking TB-IRIS, with the recognition that new TB diagnoses are frequently made in patients who have recently commenced ART but not all have the features of unmasking IRIS [13]. Two illustrative cases of paradoxical TB-IRIS cases are presented in Fig. 2.

## PARADOXICAL TUBERCULOSIS-ASSOCIATED IMMUNE RECONSTITUTION INFLAMMATORY SYNDROME

### Incidence and risk factors

Recently reported cohort studies of adult patients in Africa and India have demonstrated high TB-IRIS incidence rates (19–57%) with the highest rates in patients with low CD4 counts [3^▪^,^15^–^17^]. A study of HIV-infected and HIV-uninfected patients with TB meningitis in India reported paradoxical reactions in 11 of 13 (84.6%) HIV-infected patients, but did not report on the temporal relationship to ART initiation [18]. A recent cohort study of 104 South African children was the first to prospectively study paradoxical TB-IRIS in children concluding that the incidence of paradoxical TB-IRIS in children was low. Paradoxical TB-IRIS occurred in 7 of 104 (6.7%) children studied [4]. A recent trial of urgent (<48 h) versus poststabilization (7–14 days) ART initiation in hospitalized children in Kenya reported IRIS incidence as a primary outcome measure, using IMPAACT definitions of IRIS. A total of 27 of 179 children had a diagnosis of suspected TB at enrolment and nine children were on TB treatment at enrolment. Eighteen patients had suspected TB-IRIS during 6 months follow up, although it is not stated whether these were unmasking or paradoxical TB-IRIS [19].

Baseline low CD4 count and high HIV viral load have been associated with an increased risk of TB-IRIS in prospective studies, including randomized controlled trials (RCTs) evaluating the optimal time for ART initiation in HIV [2]. The most recently published was a study of 478 patients in Ethiopia, starting ART at 1, 4 or 8 weeks of TB treatment, powered for mortality outcome at 48 weeks. No survival benefit was evident with ART initiation at week 1 and TB-IRIS was more frequent in patients with early versus later ART initiation, with an incidence of 15 (95% CI 9–24), 5 (95% CI 2.3–11.7) and 0 per 100 person years in those who commenced ART at 1, 4 and 8 weeks, respectively (*P* = 0.001) [20].

Two meta-analyses of RCTs on the optimal time to initiate ART following commencement of TB treatment in HIV-associated TB have reported combined relative risks of TB-IRIS of 2.19 (95% CI 1.77–2.70) and 2.31 (95% CI 1.87–2.86) for early (up to 4 weeks) versus late (8–12 weeks) ART initiation [5,6]. In patients with a CD4 count below 50 cells/mm^3^, a mortality benefit was apparent with early ART, favouring ART initiation within 2 weeks following TB treatment in such patients. However, a relatively high incidence of TB-IRIS can be expected in these patients who commence ART at 2 weeks of antituberculous therapy – the risk was elevated over two-fold with early ART.

Our cohort study followed 47 TB patients (with baseline CD4 < 200 cells/μl) commencing ART, investigating immunopathology caused by matrix metalloproteinases [3^▪^]. The incidence of paradoxical TB-IRIS was 57%. Patients who went on to develop TB-IRIS had higher plasma MMP-8 and procollagen III N-terminal propeptide (PIIINP, a matrix degradation product released during collagen turnover) and evidence of an increased systemic inflammatory response (higher heart rates and higher respiratory rates) but lower lymphocyte counts, at TB diagnosis, compared to those who did not develop TB-IRIS. Urine lipoarabinomannan (LAM, a marker of renal TB indicating disseminated infection in patients with advanced HIV) was more often positive in TB-IRIS patients (adjusted odds ratio = 10.9). Marais *et al.*[7^▪^] reported increased proportions of cerebrospinal fluid (CSF) culture positivity and persistently positive CSF cultures in patients who developed TBM-IRIS relative to those who did not. However, a recent study of 90 patients with baseline CD4 count less than 250 cells/mm^3^, starting ART, nested within an RCT of early versus late ART, did not find an association between positive blood cultures for TB and TB IRIS diagnosis [21].

The question of whether integrase inhibitors increase the risk of paradoxical TB-IRIS as they result in a more rapid decline in HIV viral load has arisen with increasing use of integrase inhibitors in first-line ART regimens. Observational studies have reported an association with all-cause IRIS [22,23]. A recent meta-analysis addressing Dolutegravir use did not find an association with IRIS [24]. Clinical trials have not demonstrated an increased risk of TB-IRIS [25,26]. This question is being further addressed in ongoing trials.

In summary, paradoxical TB-IRIS frequently complicates ART initiation in HIV-associated TB, with a high incidence in selected patients. More advanced immunosuppression, more inflammatory TB presentation, disseminated TB and persistent culture positivity, and a shorter time to ART initiation from TB treatment have all been associated with increased TB-IRIS risk and together suggest that increased mycobacterial burden permitted by advanced immunosuppression is causally associated with the development of TB-IRIS.

### Outcomes

Overall mortality from paradoxical TB-IRIS appears to be relatively low but may be confounded by underdiagnosis and lack of reporting [2]. Recent cohort studies have reported few or no deaths due to TB-IRIS. The most recently published RCT of timing of ART initiation in TB patients reported only one TB-IRIS case among 64 deaths [20]. However, in a pooled analysis of RCTs that reported TB-IRIS events, the estimated relative risk of death from paradoxical TB-IRIS in early ART arms versus late arms was 6.94 (95% CI 1.26–38.22) with an event rate of 0.78% (9 of 1153 cases) in the early arms versus 0 of 1119 in the late arms [5]. Central nervous system TB-IRIS carries a particularly high mortality risk, up to 30% [7^▪^,^8^]. In our cohort study of 47 patients, hospital admission was required in 13 of 29 (45%) TB-IRIS patients compared to 1 of 18 (6%) non-IRIS controls (*P* = 0.007) [3^▪^]. There were no deaths from TB-IRIS in this study, performed in an experienced research centre with close clinical monitoring.

### Diagnostic and prognostic biomarkers

In a prospective observational study of 170 TB patients with CD4 counts less than 125 cells/μl, starting ART in Botswana, 33 (19%) patients developed paradoxical TB-IRIS and 18 (11%) patients died, but there was only one (3%) death among TB-IRIS patients, reported as occurring after the resolution of TB-IRIS symptoms [17]. This study evaluated 26 biomarkers in plasma by Luminex, in baseline pre-ART samples with respect to an eventual diagnosis of paradoxical TB-IRIS or death. Lower growth factor and Th1 cytokine responses, and lower concentrations of the proinflammatory cytokine interleukin-17 (IL-17) were associated with TB-IRIS, whereas higher baseline proinflammatory cytokines, tumour necrosis factor-α (TNF-α) and interleukin-6, in addition to MCP-1 and EOTAXIN, were associated with death, suggesting different pathophysiological mechanisms leading to TB-IRIS and early mortality due to TB, following ART initiation.

A study of 267 patients nested within the CADIRIS trial evaluated 20 biomarkers at ART baseline to investigate an association with all-cause IRIS [27]. There were 62 IRIS cases diagnosed using ACTG definitions, of which 19 (34%) were TB-IRIS (14 paradoxical, five unmasking). TB-IRIS was associated with a distinct biosignature comprising elevated C-reactive protein, soluble CD14 and interferon-γ (IFN-γ) and lower haemoglobin. These factors were combined into a score that allowed prediction of TB-IRIS versus no-IRIS with a sensitivity of 71.4% (0.52–88.7) and a specificity of 73.2% (57.1–85.9).

A Malaysian study comparing a combined group of patients with paradoxical and unmasking TB-IRIS (*n* = 15) to controls with TB but no IRIS (*n* = 14) and those without TB or IRIS (*n* = 15), using INSHI definitions, found elevated IL-18 and CXCL-10 levels prior to ART to be predictive of TB-IRIS [28]. In a validation cohort in India, baseline IL-18 predicted paradoxical TB-IRIS with an AUC = 0.742, *P* = 0.004 (no confidence intervals reported) [28]. One study investigated the predictive value of antigen-specific cytokine responses for TB-IRIS prediction and found it to be of little value [29].

### Management

Management of paradoxical TB-IRIS includes investigations to rule out other diagnoses (e.g. sepsis), supportive management (e.g. IV fluids for hypotension), symptomatic treatment (e.g. analgesia and antiemetics), surgical or percutaneous interventions (e.g. abscess drainage) and inhibition of excessive immune responses (corticosteroids). It is critical to exclude drug-resistant TB, which is an important cause of clinical deterioration in HIV-associated TB, and which can manifest with TB-IRIS features that are clinically indistinguishable from IRIS seen in drug-sensitive TB [30]. ART interruption is not recommended.

Corticosteroids remain the only treatment for paradoxical TB-IRIS whose use is supported by RCT data [11]. Other immunomodulatory therapies have been the explored in case reports and case series including several reports of anti-TNF-α therapy, and one report of intravitreal anti-VEGF (Bevacivumab) therapy in ocular TB-IRIS [31–34]. Prednisone is not licensed for the treatment of TB-IRIS and other immunotherapies are investigational.

### Prevention

Two double-blind randomized, placebo-controlled studies have assessed strategies to prevent TB-IRIS in adults. The CADIRIS study assessed the efficacy of the CCR5 blocker maraviroc in reducing all-cause IRIS, including TB-IRIS, in patients with CD4 count less than 100 cells/μl [35]. Time to an IRIS event by 24 weeks was the primary outcome. No difference in proportion of TB-IRIS was found between the maraviroc and the placebo arm.

The PredART trial assessed the efficacy and safety of prophylactic prednisone in preventing TB-IRIS in patients who were identified as being at high risk for paradoxical TB-IRIS [12^▪▪^]. Inclusion criteria included CD4 count less than 100 cells/μl, microbiologically confirmed TB or clinical diagnosis with symptomatic response to antituberculosis treatment, and starting ART within 30 days after starting antituberculosis treatment [12^▪▪^]. Exclusion criteria included Kaposi's sarcoma, neurological or pericardial tuberculosis, rifampicin-resistant tuberculosis and hepatitis B surface antigen positivity. The primary endpoint was the development of paradoxical TB-IRIS (according to the INSHI consensus definition) within 12 weeks after starting ART adjudicated by an independent committee. Two hundred and forty participants were randomized 1 : 1 to receive either prednisone (40 mg daily for 2 weeks, followed by 20 mg daily for 2 weeks) or identical placebo within 48 h of starting ART. Prophylactic prednisone reduced the risk of paradoxical TB-IRIS by 30% (56 of 120 in the placebo arm versus 39 of 120 in the prednisone arm), corresponding with an absolute reduction in incidence of 14.2%.

Prednisone was safe, with no statistically significant difference in grade 4 adverse events, severe infections, malignancies, death, corticosteroids side-effects or immunological and virological outcomes at week 12, between the prednisone and the placebo arm. There did not appear to be ‘breakthrough cases’ when the 28-day course of prednisone was stopped. Prednisone also reduced the incidence of more severe TB-IRIS, judged from the number of patients with TB-IRIS fulfilling at least one INSHI major criterion or prescribed prednisone treatment of TB-IRIS; proportions for both of these secondary outcomes were also reduced in the prednisone arm.

Further study is required to conclude whether prednisone would benefit patients not included by the enrolment criteria. Prednisone is neither licensed for treatment nor prevention of TB-IRIS. However, based on this result, we recommend that preventive treatment with prednisone, at the doses above, for HIV-infected TB patients with a CD4 nadir less than 100 cells/μl, who have had hepatitis B and Kaposi's sarcoma excluded, who are not diagnosed with rifampicin-resistant TB and who are symptomatically improving on TB treatment prior to ART. A similar recommendation has been included in the 2017 European AIDS Clinical Society guidelines [36].

### Pathogenesis

Recent evidence has implicated exaggerated cytotoxic responses, excessive proinflammatory innate immune responses mediated by inflammasome activation and MMP-driven tissue damage in TB-IRIS pathogenesis. These processes are likely to be inter-related. A detailed review of the pathogenesis of TB-IRIS is beyond the scope of this review. However, here we detail key recent studies that have enhanced our understanding.

We investigated transcription profiles of Mtb-stimulated peripheral blood mononuclear cells of TB-IRIS and controls who did not develop TB-IRIS, finding cytotoxic mediators perforin and granzyme B to be among the top differentially regulated genes [37]. Correspondingly granzyme B was increased in serum of TB-IRIS patients but reduced in prednisone-treated patients. We found elevated CD3 + Vα24+ cell populations in TB-IRIS patients suggesting that natural killer T cells may play a role in TB-IRIS. A previous report found that increased natural killer (NK) cell degranulation predicted TB-IRIS and together these studies support a role for aberrant cytotoxic responses [38]. Further studies exploring the clinical implications of these findings are required.

Lai *et al.*[39] performed transcriptomic analysis of whole blood from 17 TB-IRIS patients and 15 non-IRIS controls in a longitudinal study, pre-ART, at 0.5 weeks post-ART initiation and at 2 weeks post-ART initiation (time of IRIS onset). The early pre-IRIS transcriptomic signature on ART in patients who developed TB-IRIS was enriched for genes associated with innate immunity, including the JAK family of kinases involved in IL-6 signalling pathways, interferon signalling, pattern recognition receptors and macrophage function. At the time of TB-IRIS onset, TLR receptor, TREM-1 signalling and the role of pattern recognition receptors in recognition of bacteria and viruses were among the most upregulated pathways indicating innate immune function to be at the centre of divergent immune responses. This was validated by plasma measurement of cytokines at the time of TB-IRIS onset: IL-12p40, IL-6, TNF-α and IFN-γ were found to be significantly increased compared to non-IRIS controls. Increased IL-1β, IL-1α, caspase-1 and caspase-5 secretion from heat-killed H37Rv Mtb-stimulated PBMC from TB-IRIS patients compared to non-IRIS controls suggested increased inflammasome activation in TB-IRIS.

Excessive MMP activity is implicated in inflammatory disease in TB-IRIS and may be corticosteroid modulated [40]. In our recent study, plasma MMP-8 (neutrophil collagenase) was most significantly elevated in TB-IRIS patients, at TB diagnosis and at the time of TB-IRIS onset, compared to controls [3^▪^]. Plasma MMP-8 correlated with peripheral blood neutrophil count, suggesting that it may be neutrophil-derived. PIIINP was elevated in TB-IRIS patients at TB diagnosis and at the time of TB-IRIS. The previously described Botswanan cohort study found increases in plasma MMP-8 on ART to be associated with TB-IRIS, and abnormal pulmonary function following TB treatment, although intervention studies are required to prove a causal link [16].

Neutrophils are elevated, activated and found at the site of caseous necrosis in human TB-IRIS [10^▪^]. In transcriptional analysis of unstimulated PBMC from TB-IRIS patients, the most upregulated transcripts in TB-IRIS patients versus controls implicated increased neutrophil activity (S100A9, NLRP12, COX-1 and IL-10), and this was supported by elevated neutrophil elastase and human neutrophil peptides 1–3 in plasma of TB-IRIS patients. These data suggest that neutrophil influx to the site of disease, activation and early cell death occur in TB-IRIS patients leading to local necrosis and tissue destruction.

In the context of advanced HIV-1 and Mtb infection, impaired Mtb antigen presentation by antigen presenting cells may permit excessive mycobacterial replication pre-ART. This may be contributed to by direct effects of the HIV-1 virus and poor T-cell help. Differential recovery of T-cell subsets, particularly with respect to activation and memory cell responses, has been associated with TB-IRIS [41,42]. However, the studies described above highlight the critical role for early innate immune responses in the excessive inflammation underlying TB-IRIS. Inflammasome activation and MMP activity are potential targets for future host-directed therapies.

## UNMASKING TUBERCULOSIS-ASSOCIATED IMMUNE RECONSTITUTION INFLAMMATORY SYNDROME

Although ART has a major impact on reducing the risk of TB in HIV-infected individuals, TB remains the most frequently diagnosed opportunistic infection after ART initiation, particularly in high TB burden settings. The risk of developing TB is highest within the first 3 months after starting ART [43]. There may be delayed immune recovery after initiation of ART, increasing the period of high risk for HIV-associated TB. In addition, there may be subclinical TB (sputum culture positivity in asymptomatic individuals), prevalent in resource-limited settings such as sub-Saharan Africa where current screening tools perform suboptimally for active case finding [44,45]. Thus, a substantial number of patients, and particularly those with more advanced immunodeficiency, are at high risk for active TB after the initiation of ART because of both failure of diagnosis of prevalent TB prior to starting ART and ongoing persistent immune defects. The diagnosis of ‘unmasking TB-IRIS’ is reserved for a subgroup of patients with ART-associated TB who manifest an acute inflammatory form of TB following ART initiation (see Fig. 1 and definitions given above) [46].

In the absence of specific diagnostic tests and a robust clinical case definition to distinguish unmasking TB-IRIS from other forms of ART-associated TB, there are limited published data on its clinical manifestations. The reported incidence of unmasking TB-IRIS in South Africa and Uganda ranges between ∼1 and 6% [47–50]. In a large prospective study in Kwazulu-Natal, South Africa, unmasking TB-IRIS occurred in 19 out of 498 (3.8%) patients at a median of 12 days (IQR 7–49) after initiating ART [48]. This timing is consistent with other studies, where the onset has ranged between 4 and 79 days [47,51]. The clinical phenotype has been most frequently characterized by lymphadenitis, abscess formation (including in the central nervous system), serositis and pulmonary infiltration [47,50,51]. In the Kwazulu-Natal cohort, 3 of 25 (12%) deaths and 7 of 65 (11%) hospitalizations were attributed to unmasking TB-IRIS [48]. Risk factors identified for unmasking TB-IRIS include more advanced immunosuppression, a more pronounced response to ART (greater decline in HIV viral load and larger increase in CD4 cells), intrathoracic adenopathy on pre-ART chest radiograph, anaemia, weight loss, low BMI and elevated C-reactive protein [47,48,51].

Investigations to confirm the diagnosis of TB should be undertaken in all cases of suspected ART-associated TB, including drug susceptibility testing. It is important to exclude other causes of clinical deterioration on ART, such as other opportunistic infections and malignancies, and adverse drug reactions. There is no specific therapeutic intervention for unmasking TB-IRIS, but continuing ART, providing symptomatic treatments alongside antituberculous therapy and managing complications are key to management [51]. Paradoxical reactions may complicate the clinical course of patients with unmasking TB-IRIS after they have commenced TB treatment [52]. The routine use of corticosteroids is not recommended, but sometimes corticosteroids are used if there are severe inflammatory manifestations. There is no clinical trial evidence to support this strategy.

Despite having similar clinical presentations, there is some suggestion that the immunopathology associated with unmasking TB-IRIS may differ from the better-characterized paradoxical form. In one recent study, transcriptomic profiling of TST biopsies from three patients with unmasking TB-IRIS showed increased expression of genes in the Th2 pathway compared to both HIV-negative and HIV-positive controls without TB-IRIS. These findings were supported by increased transcriptional expression and immunostaining of interferon regulatory factor 4, which has been associated with Th2 responses [53]. Similarly to paradoxical TB-IRIS where the primacy of Th1 responses has been questioned, another study reporting a well-characterized patient with unmasking TB-IRIS showed that tuberculin-specific Th1 responses became expanded only after resolution of IRIS symptoms, and that there was a distorted balance of T-cell phenotypes favouring the central memory T-cell compartment prior to IRIS onset [54]. The role of innate immune effectors, including NK cells, in the pathogenesis of unmasking TB-IRIS has also been highlighted [55]. Additional investigations into the immunological mechanisms are needed, particularly with regard to the role of the innate immune response and the inflammasome, to identify potential predictors and therapeutic targets for unmasking TB-IRIS [9].

## FUTURE DIRECTIONS

Major insights into TB-IRIS pathogenesis have come from human observational studies with ex-vivo analysis of immune parameters. An appropriate animal model has been lacking, although a mouse model of MAI IRIS has been used [56]. A macaque SIV-Mtb coinfection model using detailed PET-CT imaging to study early immunological changes in the lung is providing useful pathophysiological insights into early HIV-TB coinfection events [57,58]. This model could in future aid in the study of TB-IRIS, particularly in attributing causality to immune mediators and pathways associated with TB-IRIS in human studies. TB-IRIS pathogenesis studies have identified multiple targets for which biological modulators exist. There is a rationale for moving more candidates to human experimental medicine studies and early-stage clinical trials. Even where an interventional study does not show a positive outcome, the pathophysiological insights provided may be extremely valuable. Strategies for future evaluation also include the use of higher doses of corticosteroids for prevention and the use of biomarkers to target preventive and treatment strategies at those most likely to benefit.

## CONCLUSION

TB-IRIS causes significant morbidity in resource-limited settings, and mortality risk may be underestimated. A sensitive and specific diagnostic test is lacking, but would be extremely valuable. Biomarkers of risk could aid in identifying high-risk patients for interventions. Clinicians should consider prednisone for TB-IRIS prevention in selected patients. Future research should focus on improving diagnosis and investigating novel therapeutic interventions.

## Acknowledgements

None.

### Financial support and sponsorship

N.F.W. is supported by a National Institute for Health Research Academic Clinical Lecturership, The British Infection Association and a Starter Grant for Clinical Lecturers (The Academy of Medical Sciences UK, Wellcome, Medical Research Council UK, British Heart Foundation, Arthritis Research UK, Royal College of Physicians and Diabetes UK). S.W. is supported by EDCTP (TMA 2015 CDF-1018) and Wellcome (203135/Z/16/Z). R.J.W. is supported by The Francis Crick Institute which receives support from UKRI (10218), Wellcome (10218) and CRUK (10218). He also receives support from Wellcome (104503, 203135) and NIH (U01A1115940). G.M. was supported by Wellcome (098316 and 203135/Z/16/Z), the South African Research Chairs Initiative of the Department of Science and Technology and National Research Foundation (NRF) of South Africa (Grant No 64787), NRF incentive funding (UID: 85858) and the South African Medical Research Council through its TB and HIV Collaborating Centres Programme with funds received from the National Department of Health (RFA# SAMRC-RFA-CC: TB/HIV/AIDS-01-2014). The funders had no role in the writing of this review. The opinions, findings and conclusions expressed in this article reflect those of the authors alone.

### Conflicts of interest

There are no conflicts of interest.

## REFERENCES AND RECOMMENDED READING

Papers of particular interest, published within the annual period of review, have been highlighted as:

▪ of special interest
▪▪ of outstanding interest

## Figures and Tables

**Table 1:** Paradoxical tuberculosis-associated immune reconstitution inflammatory syndrome – knowledge summary

Knowledge summary		Key references
Incidence	Adults overall: 18% (95% CI 16–21%), with a range of 4–54%; higher rates in patients with lower CD4 counts (up to 57% in patients with CD4 count <200 cells/μl).	Reviewed in [2] Recent cohort described in [3^▪^]
	South African children: 6.7% reported in a recent prospective study	[4]
Risk factors	Low CD4 count at ART initiation; High HIV viral load at ART initiation	Reviewed in [2]
	Shorter time between TB treatment initiation and ART initiation	Meta-analyses reported in [5,6]
	Disseminated TB/high mycobacterial load.	[3^▪^,7^▪^]
Clinical presentation	Systemic, pulmonary and lymph node presentations most common	Reviewed in [2]
	In a recent study, median days to symptom onset reported as 6 (range 1–23)	[3^▪^]
Mortality	All-cause mortality rate of 7% (95% CI 4–11%) and IRIS-attributable deaths of 2% (95% CI 1–3%)	Reviewed in [2]
	Higher mortality in CNS TB-IRIS	Reviewed in [8]
Pathogenesis	Innate immune cell activation, including neutrophils, monocytes and NK cells; Antigen-specific upregulation of cytotoxic mediators Inflammasome activation; Hypercytokinaemia (including IL-1β, IL-6 and TNF-α) and MMP upregulation/secretion	Reviewed in [9]; see also, [3^▪^,7^▪^,10^▪^]
Treatment	Prednisone (1.5 mg/kg for 2 weeks followed by 0.75 mg/kg for 2 weeks) for treatment of paradoxical TB-IRIS reduced length of hospital admission and number of therapeutic procedures required, and improved symptoms in paradoxical TB-IRIS	Randomized-controlled trial reported in [11]
	Consensus is not to stop ART, but to investigate fully for alternative causes, and provide symptomatic treatment	Reviewed in [9]
Prevention	Prednisone (40 mg daily for 2 weeks, followed by 20 mg daily for 2 weeks) from ART initiation reduces the risk of future paradoxical TB-IRIS by 30%	[12^▪▪^]
	Do not delay ART initiation beyond 2 weeks after TB treatment initiation in patients with CD4 count <50 cells/mm^3^, unless CNS TB diagnosed (then delay 4–8 weeks). Early ART improves survival in patients with CD4 < 50 cells/mm^3^ even though it increases TB-IRIS risk > two-fold	Meta-analyses reported in [5,6]
